# Determination of organic pollutants in *Anguilla anguilla* by liquid chromatography coupled with tandem mass spectrometry (LC-MS/MS)

**DOI:** 10.1016/j.mex.2021.101342

**Published:** 2021-04-15

**Authors:** Dyana Vitale, Yolanda Picó, Rodrigo Álvarez-Ruiz

**Affiliations:** Environmental and Food Safety Research Group (SAMA-UV), Desertification Research Centre (CIDE), Universitat de València-CSIC-GV, Moncada-Naquera Road km 4.5, Moncada, Valencia 46113, Spain

**Keywords:** QueChERs, EMR-lipid, Emerging pollutants, Liver, Muscle, Pesticides, Pharmaceuticals, PFASs, Illicit drugs

## Abstract

One of the aspects considered about the presence of contaminants in the aquatic ecosystems is their possible effect on critically endangered species, as the case of European eel, *Anguilla anguilla*. However, there is a lack of analytical methods to determine these contaminants due to the complexity of eel matrix (contains 5–20 % of lipids and 5–15 % of proteins). Thus, a multi-residue method using QuEChERS extraction a clean-up based on new specific sorbents (to eliminate lipids) and liquid chromatography tandem mass spectrometry (LC-MS/MS) was developed to determine a mix of 21 contaminants. Compared to the previously reported methods (Degani et al., 1986), which were developed for mussels, in this study, one of the proposed extraction methods were adapted to different fish tissues of higher complexity, such as liver and muscle of *A. anguilla*.

• The effectivity of dispersive solid phase extraction (dSPE) using new specific Enhanced Matrix Removal (EMR-lipid) as clean-up for lipid removal was tested.
• Clean extracts of matrices with high protein (5–15 %) and lipid (5–20 %) content were obtained ensuring robustness and durability of the analytical systems.
• Emerging contaminants extractable by this procedure comprise four different families (pesticides, perfluoroalkyl substances (PFASs), pharmaceuticals and drugs of abuse). Then, it could be further applied to wide scope screening strategies.

## Graphical abstract

**Figure:**
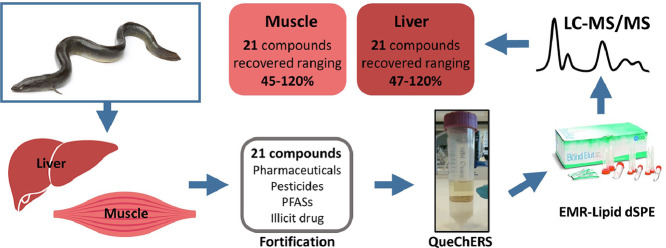

**Table:** Specifications Table

**Subject Area**	Environmental Science
**More specific subject area**	Advanced mass spectrometric analysis for environmental and food safety
**Method name**	Multi-residue method based on quick easy cheap effective rugged and safe (QuEChERS) procedure and Enhanced Matrix Removal (EMR-lipid) clean-up.
**Name and reference of original method**	Development of multi-residue extraction procedures using QuEChERS and liquid chromatography tandem mass spectrometry for the determination of different types of organic pollutants in mussel
**Resource availability**	Under review at Analytical and Bioanalytical chemistry

## Background

In Europe, *Anguilla anguilla* is critically endangered because it is highly affected by several anthropic pressures, such as habitat modification, overfishing and contamination. This latter has been widely reported in its habitats. This together with a complicated live cycle that involves migration (> 5000 km), the success of which depends on nutritional and sanitary state of the eel has caused the decline of its population. Organic pollutants have already been profusely described in any type fish [6]). The evaluation of the presence of organic pollutants, in eels is crucial to assess their possible influence in the decline of this endangered species. Eels are rich in proteins (5–15% w/w) and highly unsaturated lipids (5–20% w/w) [1]. Therefore, their analysis is also crucial to assess any risk for human health. For these tasks, the development of analytical methods capable of dealing with complex matrices (high content in proteins and lipids) is needed. In this way, multi-residue methods allow the analysis of compounds from different families at the same time, saving time and resources.

## Method details

The Multi-residue extraction and clean-up selected for this study, was originally reported in Álvarez‐Ruiz et al. [4] for mussel matrix as one of the three best methods among 44 different combinations of QuEChERS (including acidified QuEChERS) and clean-ups. The application of a novel sorbent (EMR-Lipid) based on size exclusion and hydrophobic interaction. Offers a very promising solution for the removal of the high lipid content of eel tissues. Therefore, in the present work the method was employed for the extraction of organic pollutants from eel´s muscle and liver. Furthermore, since the amount of water employed during the QuEChERS procedure could influence the method performance ([4] ), five variations of water addition have been tested. The compounds analyzed were 5 pesticides, 5 PFASs, 10 pharmaceuticals and 1 illicit drug, as in the original work, they were selected due to their presence in aquatic environments [4].

## Materials and reagents

The high-performance liquid chromatography (HPLC) grade acetonitrile (ACN) ≥ 99.9% purity and trisodium citrate dehydrate (TCD) were purchased from VWR Chemicals® (Radnor, Pennsylvania). Magnesium sulphate and disodium hydrogen citrate sesquihydrate (DCS) were from Alfa Aesar (Karlsruhe, Germany). Sodium chloride from Sigma-Aldrich (Steinheim, Germany). EMR-Lipid clean-up dSPE was from Agilent Technologies. Polypropylene centrifuge falcon tubes (either 15 mL or 50 mL) were purchased from VWR International Eurolab (Barcelona, Spain). Polypropylene/polyethylene syringes manufactured by BRAUN and distributed by Scharlab S.L., Barcelona, Spain. Nylon 0.22 µm filters were purchased from Membrane Solutions (Plano, TX, USA). The 2 mL amber glass vials with stoppers 99 mm + Septum Sil /PTFE used to inject the samples were from Análisis Vínicos S.L. (Tomelloso, Spain) and the 250 µL polypropylene inserts were from Agilent Technologies (Santa Clara, CA, United States).

The analytical standards of pharmaceuticals (acetaminophen, atenolol, caffeine, diclofenac, etoricoxib, ibuprofen, naproxen, salicylic acid, triclosan, vildagliptin), pesticides (bentazone, chlorfenvinphos, chlorpyrifos, imazalil, terbutylazine), and PFASs (perfluoropentanoic acid [PFPeA] and perfluorobutanesulfonate [PFBS]) were from Sigma-Aldrich (Steinheim, Germany). While perfluorooctanoic acid (PFOA), perfluorodecanoic acid (PFDA), and perfluorooctanesulfonate (PFOS) were from Wellington (Ontario, Canada) and the illicit drug bufotenine was purchased from LGC Standards (Ontario, Canada). The surrogate (internal) standards acetaminophen-d3 and atenolol-d7 were from Sigma– Aldrich. Chlorfenvinphos-d10 (diethyl D5), chlorpyrifos-d10 (diethyl D10), and vildagliptin-d3 were purchased from LGC Standards. Diclofenac-d4 was purchased in Toronto Chemicals Research (Toronto Canada). PFOA-d4 (MPFOA), PFOS-d4 (MPFOS), and PFDA-d4 (MPFDA) were from Wellington. Both the internal and external standard mix were created using ACN as solvent.

## Sampling

For this study, approximately 25 *A. anguilla* specimens were obtained from a fish local market and supermarket at, Valencia (Spain), then refrigerated and transferred to the laboratory of Food and Environmental Safety Research Group (SAMA-UV), Desertification Research Centre (CIDE, UV-CSIC-GV), University of Valencia, Spain. Once in the laboratory, samples of liver and muscle tissue were pooled. Muscle was chopped in small pieces and then homogenize using a pestle and placed in 50 mL Falcon tubes. Since the livers are soft and a very scarce and valuable resource (1,2 g for specimen), they were chopped, placed in a 50 mL falcon tube (to avoid any loss of sample) and then homogenized using metal tweezers. The tubes were then stored at -20 °C until analysis.

## Extraction procedure (QuEChERS)

An aliquot of 1 g w.w. (wet weight) of pooled eel liver or muscle placed in 50 mL falcon tubes was spiked with 200 µL of an internal standard mix at 1 mg/mL to achieve a final concentration in the extracts of 20 ng/mL (assuming a recovery of the 100%). Also 50 µL of the external standard mix at 10 mg/mL (what is translated in to 500 ng/g) were added and the sample was left until the solvent evaporated. Water was added and to ensure the optimal partitioning 0, 3, 5, 7.5, and 10 mL were tested. Then, ACN (10 mL) was also added. The mix was vortexed for 3 min to ensure homogenization. Next, 4 g of MgSO_4_, 1 g of NaCl, 0.5 g of DCS and 1 g of TCD were added and, immediately, the tube was vigorously shaken by hand for 3 min to avoid salt agglomeration and then, centrifuged for 5 min at 3500 rpm (2465 rcf).

For the clean-up, EMR-Lipid dSPE phase was placed in 15 mL Falcon tubes and activated by adding 5 mL of MilliQ water and vortexed for 30 s. Then, 5 mL of QuEChERS supernatant were added, the tube was vortexed for 30 s more and centrifuged for 5 min at 3500 rpm. Next, 5 mL of this supernatant were added to another 15 mL Falcon tube containing the polish phase consisting of a mixture of 1600 mg of MgSO_4_ and 400 mg of NaCl. The tube was manually shaken for 30 s to avoid salt agglomeration and then, centrifuged for 5 min at 3500 rpm. The supernatant was filtered using Nylon 0.22 µm filters and polypropylene/polyetilene syringes, and then, stored in vials with inserts ready for analysis.

Recovery tests were performed in triplicate for each variation in the water amount. Furthermore, a procedural blank containing non-spiked sample was included at least every 9 samples.

## LC-MS/MS analysis

Analysis was performed via LC-MS/MS as described by Álvarez‐Ruiz et al. [5], an Agilent 1260 UHPLC from Agilent technologies coupled to an Agilent 6410 Mass Spectrometer triple quadrupole (QqQ) also from Agilent technologies were employed. With electrospray source ionization (ESI) in both negative and positive ionization modes (nebulizer gas 15 psi, gas flow 11 L/min. ion-spray voltage 4 kV and temperature 300 °C) operated in multiple reaction monitoring mode (MRM). The column used for the detection of pesticides and etoricoxib was Luna® 3 µm C18(2) 100 Å 150 × 2 mm and the column employed for PFAS, illicit drug and the rest of PPCPs was a Kinetex 1.7 µm XB-C18 100 Å 50 × 2.1 mm, both from Phenomenex. Yielding a total of three LC methods, one in negative ionization mode using Kinetex column and two in positive mode using Kinetex and Luna® columns, respectively. When operated in positive ionization mode, the mobile phases employed were (A) H_2_O 0.1% formic acid and (B) MeOH 0.1% formic acid. For negative ionization mode, the mobile phases employed were (A) H_2_O 2.5 mM ammonium fluoride and (B) MeOH 2.5 mM ammonium fluoride. The linear gradient was as follows: 0 min (70% A), 12 min (5% A), 25 min (5% A), 26 min (70% A), and 30 min (70% A) either in positive or negative ionization mode (only the mobile phases were different). The column temperature was 30 °C. The injection volume was 5 µL and the ACN extract were directly injected. As the injection volume is low, no modification of the retention times was observed for pesticides and PFASs. However, the pharmaceuticals and illicit drugs some retention times are shorter but not double peaks were observed. Detailed information on the retention times, the selected transitions (precursor ion → product ion) for each compound, and those compounds that were determined using internal standards as well as by external standard is available in the Supplementary material (Table S1). Information on the transitions used to determine the internal standards are also reported in Supplementary material (Table S2). The confirmation of the presence of a target compounds in the sample was carried out considering the presence of the two transitions (precursor ion → product ion) and relative intensity of the two product ions (if possible), and retention time. As can be observed except for PFPeA and Ibuprofen that only gave one transition with enough intensity, for the other compounds two transitions were selected in order to ensure a proper identification. Data were obtained using the Mass Hunter software, qualitative (for the identification of substances) and quantitative (for the quantity of the substances obtained on time). Each batch of samples included a procedural blank (non-spiked tissues pool). At the beginning and at the end of each analytical sequence, a seven-points calibration standard was injected. A 100 ng/mL standard was also injected every 15 samples to check the instrumental variation and to avoid false negatives as well solvent and procedural blanks were injected to avoid false positive.

## Method optimization

**Fig. 1:**
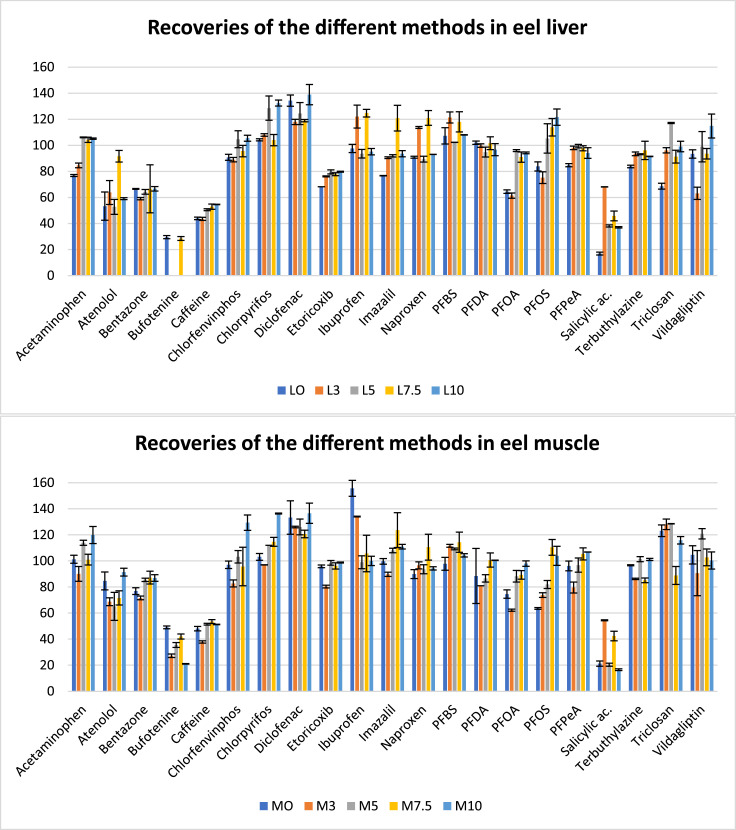
Recoveries of the 5 methods with different addition of water tested (“L” for liver tissue and “M” for muscle with 0;3;5;7,5:10 ml of water). The compounds with internal standard are represented in RR%, while the rest of compounds are represented in E%. Error bars are set as ± SD.

The addition of different volumes of water was tested in order to optimize the partition between water and ACN that takes place in QuEChERS after salting out. Results showed not significant differences in the recoveries using different water volumes even for the most polar compounds (bufotenine, salycilic acid and caffeine) (Fig. 1). Then, the volume of 7.5 mL was chosen in order to keep proportion used in the original QuEChER.

Regarding the LC-MS/MS analysis it is possible to determine the compounds using just the Kinetex column. However, the pesticides and etoricoxib usually presented chromatograms remarkably with better signal-noise ratio and better shaped peaks when Luna® was employed. Hence both were employed, as described above, in order to obtain better LC-MS/MS information.

## Method validation

The selected protocol was validated for specificity, accuracy, precision, linearity, limits of detection (LOD), limit of quantification (LOQ), and matrix effects (ME), as described in Álvarez‐Ruiz et al. [5]. The linearity was prepared in ACN, established through seven-points calibration standards (5, 10, 25, 50, 100, 200 and 500 ng/mL) and only regression coefficients (R^2^) > 0.99 were accepted in the calibration curve. Recoveries were calculated in eel liver and muscle tissues fortified at 50, 250 and 500 ng/g in triplicate (5, 25 and 50 ng/mL the final extract). A 7-points calibration curve that considers peak areas or if the internal standard is available the ratio of the peak area and the area of the internal standards (*y*-axis) vs. contaminant concentrations (*x*-axis), Then, the peak area or its ratio with the internal standard of the sample is interpolated in the calibration curve to quantify them. For the compounds chlorfenvinphos, chlorpyrifos, PFDA, PFOA, PFOS, acetaminophen, atenolol, diclofenac and vildagliptin, the results obtained were relative recoveries (RR%) where the ME and other potential inaccuracy during sample handling, were corrected using the internal standards. The other compounds: imazalil, bentazone, terbuthylazine, PFBS, PFPeA, bufotenine, caffeine, etoricoxib, ibuprofen, naproxen, salicylic ac. and triclosan were quantified with external calibration. Hence, the results were represented as efficiency (E%), if the results are affected by either recovery and ME, or absolute recoveries (AR%), if the ME is corrected using matrix-matched standards. Both were calculated following Eq. (1).

(1) $$RR\%\;or\;E\%=\left(\frac{Final\;concentration\;of\;the\;spiked\;sample}{\text{EC}}\right)\;\cdot 100$$

where EC is the expected concentration in the final extract assuming a recovery of 100%. For the determination of ME, 7 mixes with the calibration curve concentration levels (5, 10, 25, 50, 100, 200, and 500 ng/mL) were prepared in ACN. For each concentration, 300 µL of mix was placed in 15 mL falcon tubes and blown down to dryness under a gentle stream of nitrogen and they were redissolved adding 300 µL of extract (muscle or liver) from a non-spiked sample. This extract was then vortexed 30 s, sonicated 3 min and injected. After LC-MS/MS analysis using external calibration, ME was calculated comparing the slope of the calibration curve in matrix and the slope of the calibration curve in ACN [9] (Eq. (2)).

(2) $$ME=\left(\frac{Slope\;of\;calibration\;curve\;in\;matrix}{Slope\;of\;calibration\;curve\;in\;ACN}\right)\;\cdot 100-100$$

The E% of the compounds without internal standard was corrected using the ME to obtain AR% using Eq. (3).

(3) $$AR\%=\frac{E\%}{\left(100+\text{ME}\%\right)}\cdot 100$$

Sensitivity was established as method limits of detection (LODs) and method limits of quantification (LOQs) (Table 2) by analysing the extractions fortified at 50 ng/g used for the recoveries described above. The extracts (performed in triplicate) were injected in duplicate (n = 6). LODs were set as three times the standard deviation (SD) of their signal and LOQs were set as 10 times the SD.

**Fig. 2:**
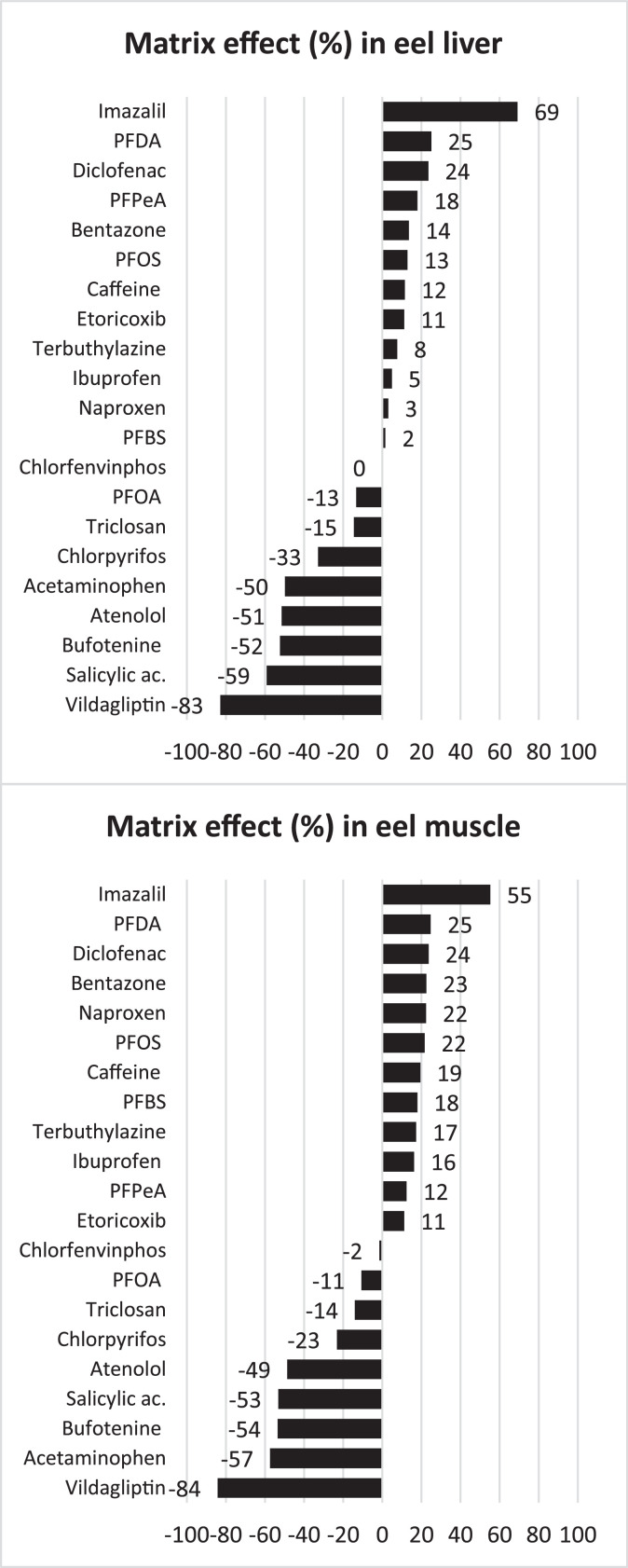
Matrix effects of the validated method in eel liver and muscle.

Due to the complexity of the matrices de ME was categorized as low (≤ ± 20%), moderate (± 20–50%) and strong (≥ ± 50%). Results showed from low to moderate ME for most of the compounds, while just 6 and 5 compounds showed strong ME in liver and muscle, respectively (Fig. 2). Strong suppression of the signal was found for atenolol, salicylic acid, bufotenine, acetaminophen and vildagliptin in both matrices. On the other hand, imazalil presented strong signal enhancement, also in both matrices. The use of isotopically labelled internal standards helps to compensate any interference, such as those from ME (signal suppression/enhancement), hence improving accuracy and precision.

Precision was evaluated in terms of repeatability (Intra-*R*) and reproducibility (Inter-*R*). Intra-*R* was calculated as the SD of the signal divided by its mean (% RSDs) of the six injections used for the determination of LODs and LOQs injected in a row. Inter-R was determined injecting one replicate of the extracts fortified at 50 ng/mL also used for the recoveries described above in three different days (n = 3). Then Inter-R was also calculated as the SD of the signal divided by its mean (% RSDs).

Intra-R was satisfactory (< 20%) except for bufotenine in liver (Table 2). Inter-*R* was also satisfactory (< 30%) except for ibuprofen and, again, bufotenine in liver. In fact, bufotenine showed to be the compound with poorer recoveries and reproducibility. LODs for liver were in the range of 1.4–9.2 ng/g except for bufotenine (11.0 ng/g), while for muscle ranged 1.5-9.2 ng/g, except for triclosan (10 ng/g). In both cases, PFPeA presented LODs of 12.00 ng/g, however, since it was not detected in the 50 ng/g spiked sample it was calculated with 3 consecutive injections of the lowest point (5 ng/mL) of the calibration curve. As well as the LODs of atenolol and bufotenine (just for muscle samples), since they were not detected in the 50 ng/g samples either.

**Fig. 3:**
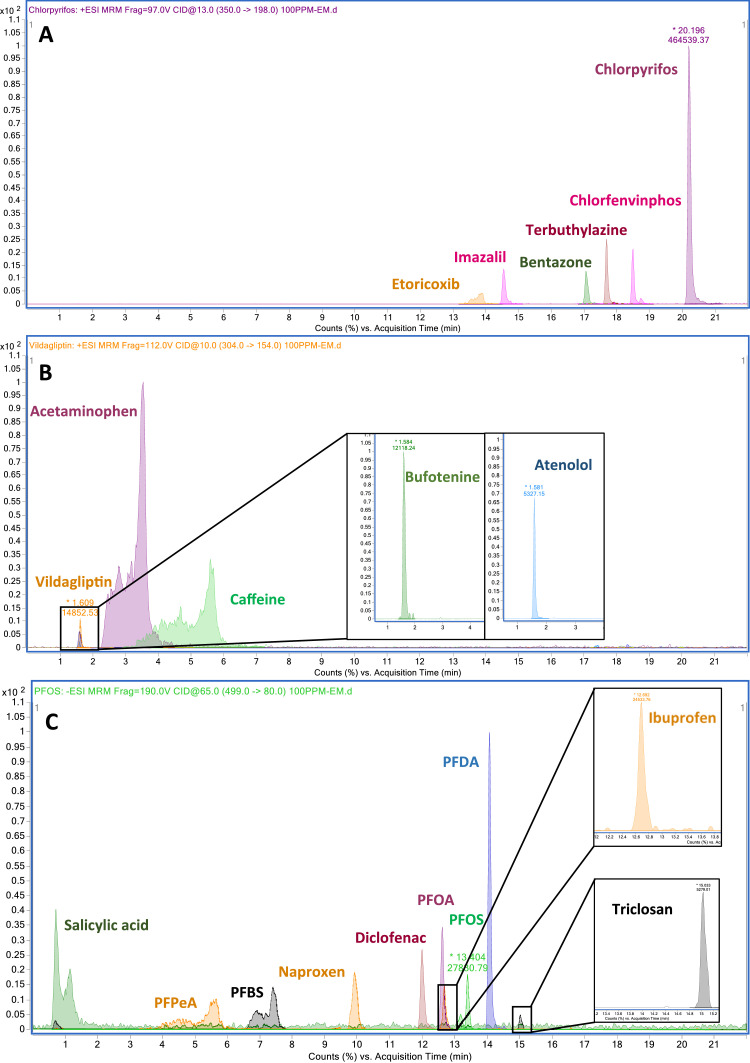
Chromatograms of the compounds analysed in the present study in an extract employed for the calculation of matrix effect in eel muscle spiked at 100 ng/mL. (A) pesticides and etoricoxib analysed in positive mode employing Luna® column. (B) Rest of the compounds analysed in positive mode employing Kinetex column. (C) Compounds analysed in negative mode.

Examples of the chromatographic peaks obtained for extracts of spiked Eels at 100 ng g^−1^ of each compound are presented in Fig. 3.

In accordance with the European Commission Guidelines, the recoveries range within 70–120% are considered acceptable [2]. However, since E% might be affected by strong ME, according to the European Commission Guidelines, also the recoveries of 50–140% were considered "satisfactory", comparing the different methods prior validation for a (RSD ≤ 20%) of individual recoveries in routine analysis [2]. In this way, for the samples spiked at 500 ng/g, 18 and 20 compounds were recovered in the acceptable range (70–120%) for liver and muscle, respectively (Table 1). Both matrices presented 20 compounds in the satisfactory (50–140%) range. Samples spiked at 50 and 250 ng/g showed similar results to those from the samples spiked at 500 ng/g for most of the compounds, with slightly poor recoveries. Bufotenine, atenolol and caffeine were the compounds with poorer recoveries (Table 1).

**Table 1:** Recoveries of the validated method in both eel liver and muscle matrix.

Recoveries (%)	Eel liver			Eel muscle		
	50 ng/g	250 ng/g	500 ng/g	50 ng/g	250 ng/g	500 ng/g
**Acetaminophen**	120	97	100	95	96	100
**Atenolol**	-	58	92	-	55	72
Bentazone	42	120	59	74	110	71
Bufotenine	69	23	60	-	-	90
Caffeine	46	64	47	50	51	45
**Chlorfenvinphos**	81	67	96	94	82	96
**Chlorpyrifos**	85	95	100	97	97	120
**Diclofenac**	93	96	120	110	96	120
Etoricoxib	90	88	70	90	89	86
Ibuprofen	140	120	120	61	81	91
Imazalil	71	77	71	55	77	80
Naproxen	100	140	120	77	79	90
PFBS	99	120	110	80	85	95
**PFDA**	89	92	100	100	98	100
**PFOA**	79	74	91	91	73	89
**PFOS**	110	110	110	110	90	110
PFPeA	-	120	83	-	110	94
Salicylic ac.	140	130	110	140	72	91
Terbuthylazine	91	97	89	75	88	73
Triclosan	79	120	110	110	130	100
**Vildagliptin**	110	89	94	81	98	100

^(a)^ Bold files represent RR%, the other compounds are represented in AR%.

^(b)^ “-“ indicates that the compound was not recovered.

**Table 2:** Validation of the selected method in terms of sensitivity (LODs, LOQs), Intra-*R* and Inter-*R*.

	Eel liver				Eel muscle			
	LOD (ng/g w.w.)	LOQ (ng/g w.w.)	Intra-R (% RSDs)	Inter-R (% RSDs)	LOD (ng/g w.w.)	LOQ (ng/g w.w.)	Intra-R (% RSDs)	Inter-R (% RSDs)
Acetaminophen	3.4	10.0	1.9	3.4	2.5	7.6	1.8	7.8
Atenolol*	9.2	28.0	5.8	3.6	9.2	28.0	5.8	17.0
Bentazone	4.7	14.0	6.5	25.0	8.9	27.0	6.5	11.0
Bufotenine*	11.0	34.0	31.0	30.0	5.9	18.0	4.2	8.4
Caffeine	1.6	4.7	2.1	7.3	1.5	4.6	1.7	5.3
Chlorfenvinphos	3.3	9.9	2.7	15.0	2.4	7.2	1.7	23.0
Chlorpyrifos	1.4	4.3	1.1	8.0	3.4	10.0	2.3	6.5
Diclofenac	3.2	9.7	2.3	1.5	2.3	6.9	1.3	4.7
Etoricoxib	5.2	16.0	3.4	2.1	5.3	16.0	3.5	5.7
Ibuprofen	4.0	12.0	1.8	32.0	5.2	16.0	4.8	27.0
Imazalil	3.4	10.0	1.9	24.0	4.8	14.0	3.7	26.0
Naproxen	3.0	9.0	1.9	20.0	7.6	23.0	5.4	18.0
PFBS	4.9	15.0	3.1	17.0	6.6	20.0	4.5	14.0
PFDA	2.9	8.8	2.2	9.6	2.8	8.3	2.1	11.0
PFOA	2.9	8.6	2.4	9.7	2.0	5.9	1.5	8.2
PFOS	4.5	13.0	2.8	21.0	5.6	17.0	3.4	11.0
PFPeA*	12.0	36.0	4.7	4.0	12.0	36.0	4.7	8.8
Salicylic ac.	7.6	23.0	8.5	16.0	6.7	20.0	6.6	18.0
Terbuthylazine	1.8	5.4	1.2	12.0	3.9	12.0	2.9	4.3
Triclosan	9.1	27.0	9.0	20.0	10.0	31.0	7.3	16.0
Vildagliptin	2.8	8.3	1.6	8.9	3.2	9.6	2.6	12.0

^(a)^ *: LODs, LOQs and Intra-*R* were calculated using the lowest point of the linearity (5 ng/ml). Except bufotenine in eel liver.

Previous studies employing QuEChERS and EMR-Lipid dSPE in liver and muscle from *A. anguilla* have not been found. A similar method was developed by [7] for the determination of niclosamide in fish, including *A. Anguilla*. The study employed HPLC and LC-MS/MS and the QuEChERS method, combining extraction and cleanup in one step. Fat content of *A. anguilla* in that study was reported to be 20.86% . This high lipid content made niclosamide more difficult to extract from eel than the other fish, the fat content also contributed to strong ME. Another study by Peña-Herrera et al. [3], reported the quantification of 21 pharmaceutical active compounds in fish muscle, using the Norwegian Atlantic salmon as matrix. They employed QuEChERS extraction and three different clean-up methods, where the EMR-Lipid dSPE yielded the best recoveries for 21 of 27 analytes and for the majority of the analytes recoveries were > 70%. The validated method was applied to natural riverine fish from the Evrotas river (Greece) and the Adige river (Italy) with positive findings for acetaminophen, propranolol, and venlafaxine reaching concentrations as high as 80 ng/g in muscle.

The validated method was tested in non-spiked samples from three different local markets, extracted by triplicate. The results showed that triclosan was detected in muscle of eels from one of the supermarkets with concentrations below the LOQs. While PFOA concentrations were also below the LOQs in muscle of eels from two markets.

In conclusion, the method validated in the present study successfully extracted a wide variety of compounds in eel liver and muscle. In the case of muscle, 20 target compounds were extracted in the range of 70–120, while liver presented 18 compounds in that range. This study in *A. anguilla species* showed to be a promising tool for future studies also in other organisms related to toxicology, metabolomics and occurrence monitoring of organic pollutants.

## Further considerations and future perspectives

The study of complex matrices sometimes implies unusual behaviors; such was the case of the chromatograms of PFOS when extracted from liver. Next to the characteristic peak of PFOS (RT 13.4) a secondary peak (RT 14) was present in every liver sample, the shape of this peak was more or less constant in all the liver samples (Fig. S1). Despite both peaks were very close, they were not overlapped, allowing the correct determination of PFOS. The formation of branched isomers in biota samples, especially in the liver due to the presence of several enzymatic pathways, has already been reported [8]. Although further study would be required, this is the most probably explanation.

The QuEChERS method involves a partition between water and ACN by salting out. The proper partition of the contaminants will depend on the water volume. Originally, the QuEChERS method was developed for pesticides (from moderate to non-polar) in vegetables. Then, vegetables have a water content ranging from 75 to 82 % and the original amount of samples was 10 g. In this case, the addition of water to the QuEChERS was not recommended. In this study, as the eel amount is 1 g, the amount of water would be 10 times lower than that present in the original method, so the addition of water is need for a proper salt partition. However, an excess of water could be disadvantaging for compounds highly soluble in water, such as pharmaceuticals, that are that might get dissolved in the water layer rather than in the can, such was likely the case of metformin in Álvarez‐Ruiz et al. [4]. For this reason, different additions of water were tested in the present work, however no significant improvement was observed for the compounds tested.

A solution to improve the LOQs might be the concentration of the extract's prior injection. However, it may imply the concentration of possible residues (protein and lipids) present in the matrix, generating important matrix interferences and also reducing the life of the columns. For this reason, no concentration step was applied in this study.

Multi-residue extraction procedures for biota are relatively scarce. The development and optimization of new methods is crucial to move forward in the analysis of these complex matrices and the protection of the biosphere. The insight and questions provided by this study shows the need to keep researching to develop more efficient and sensitive methodologies.

Supplementary material *and/or* Additional information:

Supplementary material associated with this article can be found separately.

## Declaration of Competing Interest

The authors declare that they have no known competing financial interests or personal relationships that could have appeared to influence the work reported in this paper.

## Appendix Supplementary materials

Image, application 1
