# Supplementary material for: Determination of organic pollutants in Anguilla anguilla by liquid chromatography coupled with tandem mass spectrometry (LC-MS/MS)
Source: MethodsX. 2021 Apr 15;8:101342. doi: 10.1016/j.mex.2021.101342 (PMC8374397; doi:10.1016/j.mex.2021.101342)
Supplement: Supplementary file 1 [file mmc1.docx]

**Supplementary Information**

**Determination of organic pollutants in *Anguilla anguilla* by liquid chromatography coupled with tandem mass spectrometry (LC-MS/MS)**

Dyana Vitale^[[1]](#footnote-1)^*, Yolanda Picó, Rodrigo Álvarez-Ruiz,

Environmental and Food Safety Research Group (SAMA-UV), Desertification Research Centre (CIDE), Universitat de València-CSIC-GV, Moncada-Naquera Road km 4.5, 46113 Moncada, Valencia, Spain

Table of contents

| **Table S-1:** External standards LC-MS/MS information. | S-2 |
| --- | --- |
| **Table S-2:** Mass labelled standards LC-MS/MS information. | S-3 |
| **Table S-3:** Recoveries of the 5 methods with different addition of water tested | S-4 |
| **Figure S-1:** PFOS chromatograms in spiked eel liver and muscle, and the 500 ng/mL point of the calibration curve. Liver samples present a secondary peak at T ≈ 13.8 min. which is not a characteristic peak of the PFOS. | S-5 |

**Table S-1:** External standards LC-MS/MS characteristics (transitions, retention time, and internal standard).

| **Analyte ID** | **Q1 Mass (Da)** | **Q3 Mass (Da)** | **Retention time (min)** | **Mass labelled** | **Fragmentor** | **Collision Energy** |  |  |
| --- | --- | --- | --- | --- | --- | --- | --- | --- |
| POSITIVE COMPOUNDS |  |  |  |  |  |  |  |  |
| Etoricoxib 1 | 359 | 280 | 14 |  | 181 | 30 |  |  |
| Etoricoxib 2 | 359 | 279 | 14 |  | 181 | 46 |  |  |
| Chlorfenvinphos 1 | 359 | 155 | 18.4 | Chlorfenvinphos-d10 | 120 | 10 |  |  |
| Chlorfenvinphos 2 | 359 | 127 | 18.4 | Chlorfenvinphos-d10 | 120 | 15 |  |  |
| Chlorpyrifos 1 | 350 | 198 | 20.1 | Chlorpyrifos-d10 | 97 | 13 |  |  |
| Chlorpyrifos 2 | 350 | 97 | 20.1 | Chlorpyrifos-d10 | 92 | 13 |  |  |
| Vildagliptin 1 | 304 | 154 | 1.6 | Vildagliptin-d3 | 112 | 10 |  |  |
| Vildagliptin 2 | 304 | 91 | 1.6 | Vildagliptin-d3 | 112 | 30 |  |  |
| Imazalil 1 | 297 | 201 | 14.5 |  | 120 | 20 |  |  |
| Imazalil 2 | 297 | 159 | 14.5 |  | 120 | 20 |  |  |
| Atenolol 1 | 267 | 91 | 1.5 | Atenolol-d7 | 91 | 57 |  |  |
| Atenolol 2 | 267 | 77 | 1.5 | Atenolol-d7 | 91 | 77 |  |  |
| Terbuthylazine 1 | 230 | 174 | 17.6 |  | 97 | 13 |  |  |
| Terbuthylazine 2 | 230 | 96 | 17.6 |  | 95 | 25 |  |  |
| Bufotenine 1 | 205 | 160 | 1.5 |  | 98 | 14 |  |  |
| Bufotenine 2 | 205 | 58 | 1.5 |  | 98 | 10 |  |  |
| Caffeine 1 | 195 | 138 | 5.6 |  | 109 | 18 |  |  |
| Caffeine 2 | 195 | 110 | 5.6 |  | 109 | 22 |  |  |
| Acetaminophen 1 | 152 | 110 | 3.5 | Acetominophen-d3 | 88 | 14 |  |  |
| Acetaminophen 2 | 152 | 92 | 3.5 | Acetominophen-d3 | 88 | 25 |  |  |
| Bentazone 1 | 241 | 107 | 17 |  | 76 | 0 |  |  |
| Bentazone 1 | 241 | 199 | 17 |  | 76 | 16 |  |  |
|  |  |  |  |  |  |  |  |  |
| NEGATIVE COMPOUNDS |  |  |  |  |  |  |  |  |
| Salicylic acid | 137 | 93 | 0.7 |  | 86 | 10 |  |  |
| PFDA 1 | 513 | 469 | 14 | MPFDA^a^ | 89 | 5 |  |  |
| PFDA 2 | 513 | 269 | 14 | MPFDA | 89 | 13 |  |  |
| PFOS 1 | 499 | 99 | 13.4 | MPFOS^b^ | 190 | 41 |  |  |
| PFOS 2 | 499 | 80 | 13.4 | MPFOS | 190 | 65 |  |  |
| PFOA 1 | 413 | 369 | 12.6 | MPFOA^c^ | 87 | 5 |  |  |
| PFOA 2 | 413 | 169 | 12.6 | MPFOA | 87 | 5 |  |  |
| PFBS 1 | 299 | 99 | 7.4 |  | 142 | 38 |  |  |
| PFBS 2 | 299 | 80 | 7.4 |  | 142 | 26 |  |  |
| Diclofenac sodium 1 | 294 | 250 | 12 | Diclofenac-d4 | 88 | 10 |  |  |
| Diclofenac sodium 2 | 294 | 178 | 12 | Diclofenac-d4 | 88 | 22 |  |  |
| Triclosan 1 | 289 | 35 | 15 |  | 98 | 14 |  |  |
| Triclosan 2 | 287 | 35 | 15 |  | 98 | 14 |  |  |
| PFPeA 1 | 263 | 219 | 5.6 |  | 66 | 5 |  |  |
| Naproxen 1 | 229 | 185 | 10 |  | 76 | 6 |  |  |
| Naproxen 2 | 229 | 170 | 10 |  | 76 | 15 |  |  |
| Ibuprofen 1 | 205 | 159 | 12.7 |  | 68 | 2 |  |  |

^a^MPFDA: Perfluoro-n-[1,2-^13^C2]decanoic acid

^b^MPFOS: Sodium perfluoro-1-[1,2,3,4-^13^C4]octanesulfonate

^c^MPFOA: Perfluoro-n-[1,2,3,4-^13^C4]octanoic acid

**Table S-2:** Mass labelled standards LC-MS/MS characteristics.

| **Analyte ID** | **Q1 Mass (Da)** | **Q3 Mass (Da)** | **Retention time (min)** | **Fragmentor** | **Collision Energy** |
| --- | --- | --- | --- | --- | --- |
| POSITIVE COMPOUNDS |  |  |  |  |  |
| Chlorfenvinphos-d10 1 | 369 | 101 | 18.4 | 109 | 32 |
| Chlorfenvinphos-d10 2 | 369 | 170 | 18.4 | 109 | 56 |
| Chlorpyrifos-d10 1 | 360 | 199 | 20.2 | 114 | 32 |
| Chlorpyrifos-d10 2 | 360 | 99 | 20.2 | 114 | 32 |
| Vildagliptin-d3 1 | 307 | 157 | 1.7 | 119 | 12 |
| Vildagliptin-d3 2 | 307 | 93 | 1.7 | 119 | 32 |
| Atenolol-d7 1 | 274 | 145 | 1.5 | 45 | 28 |
| Atenolol-d7 2 | 274 | 79 | 1.5 | 45 | 24 |
| Acetominophen-d3 1 | 155 | 111 | 3.1 | 96 | 14 |
| Acetominophen-d3 2 | 155 | 65 | 3.1 | 96 | 34 |
|  |  |  |  |  |  |
| NEGATIVE COMPOUNDS |  |  |  |  |  |
| MPFDA 1 | 515 | 270 | 14 | 92 | 12 |
| MPFDA 2 | 515 | 470 | 14 | 92 | 5 |
| MPFOS 1 | 503 | 99 | 13.5 | 180 | 41 |
| MPFOS 2 | 503 | 80 | 13.5 | 180 | 61 |
| MPFOA 1 | 417 | 372 | 12.6 | 82 | 5 |
| MPFOA 2 | 417 | 169 | 12.6 | 82 | 13 |
| Diclofenac-d_4_ 1 | 298 | 254 | 12 | 15 | 10 |

**Table S-3:** Recoveries obtained for the two matrices (L: liver and M: muscle) adding different amounts of water.

|  | **Recoveries (%)** | | | | | | | | | | | | | | | | | | | | |
| --- | --- | --- | --- | --- | --- | --- | --- | --- | --- | --- | --- | --- | --- | --- | --- | --- | --- | --- | --- | --- | --- |
|  | **Liver** | | | | | | | | | |  | **Muscle** | | | | | | | | | |
|  | *L0* | *SD*  *n=3* | *L3* | *SD*  *n=3* | *L5* | *SD*  *n=3* | *L7.5* | *SD*  *n=3* | *L10* | *SD*  *n=3* |  | *M0* | *SD*  *n=3* | *M3* | *SD*  *n=3* | *M5* | *SD*  *n=3* | *M7.5* | *SD*  *n=3* | *M10* | *SD*  *n=3* |
| **Acetaminophen** | 77 | 0.8 | 85 | 1.8 | 110 | 0.3 | 100 | 2.0 | 110 | 0.5 |  | 100 | 3.1 | 90 | 5.7 | 110 | 2.0 | 100 | 4.4 | 120 | 6.6 |
| **Atenolol** | 53 | 11.0 | 64 | 9.1 | 53 | 5.7 | 92 | 4.5 | 59 | 0.8 |  | 85 | 6.9 | 69 | 3.0 | 65 | 11.0 | 72 | 0.8 | 91 | 3.0 |
| Bentazone | 67 | 0.3 | 59 | 0.8 | 64 | 1.8 | 67 | 18.0 | 67 | 1.8 |  | 77 | 2.7 | 72 | 1.4 | 85 | 1.3 | 87 | 4.9 | 87 | 2.6 |
| Bufotenine | 30 | 1.2 | - | - | - | - | 29 | 1.5 | - | - |  | 49 | 1.1 | 27 | 1.4 | 36 | 1.8 | 42 | 10.0 | 21 | 0.2 |
| Caffeine | 44 | 0.9 | 44 | 1.2 | 51 | 0.7 | 53 | 2.0 | 55 | 0.1 |  | 48 | 1.7 | 38 | 0.9 | 51 | 0.7 | 54 | 1.4 | 51 | 0.2 |
| **Chlorfenvinphos** | 91 | 2.3 | 89 | 1.7 | 110 | 6.5 | 96 | 4.3 | 110 | 2.3 |  | 97 | 2.8 | 83 | 2.7 | 100 | 4.7 | 96 | 15.0 | 130 | 5.9 |
| **Chlorpyrifos** | 100 | 0.8 | 110 | 1.0 | 130 | 9.3 | 104 | 4.4 | 130 | 2.3 |  | 100 | 2.6 | 97 | 0.1 | 110 | 0.0 | 120 | 3.4 | 140 | 0.4 |
| **Diclofenac** | 130 | 4.4 | 120 | 2.0 | 120 | 8.4 | 119 | 0.9 | 140 | 7.8 |  | 130 | 13.0 | 130 | 0.6 | 130 | 6.1 | 120 | 2.9 | 140 | 7.8 |
| Etoricoxib | 68 | 0.1 | 76 | 0.4 | 80 | 1.6 | 78 | 1.1 | 80 | 0.4 |  | 96 | 1.1 | 80 | 1.0 | 99 | 1.8 | 96 | 2.4 | 99 | 0.4 |
| Ibuprofen | 98 | 3.2 | 120 | 8.8 | 94 | 3.2 | 120 | 2.9 | 95 | 2.5 |  | 160 | 6.1 | 130 | 0.3 | 99 | 4.9 | 110 | 0.1 | 100 | 3.6 |
| Imazalil | 77 | 0.2 | 91 | 0.7 | 92 | 0.9 | 120 | 9.9 | 94 | 2.4 |  | 100 | 2.1 | 90 | 1.7 | 110 | 1.7 | 120 | 13.0 | 110 | 1.7 |
| Naproxen | 91 | 0.7 | 110 | 0.8 | 89 | 2.2 | 120 | 5.7 | 93 | 0.1 |  | 90 | 3.5 | 97 | 2.7 | 94 | 3.4 | 110 | 9.9 | 94 | 1.3 |
| PFBS | 110 | 6.3 | 120 | 4.2 | 100 | 0.1 | 120 | 7.8 | 110 | 0.1 |  | 98 | 4.9 | 110 | 1.1 | 110 | 0.7 | 110 | 5.9 | 100 | 1.3 |
| **PFDA** | 100 | 1.3 | 100 | 1.3 | 95 | 3.8 | 100 | 4.9 | 97 | 4.6 |  | 88 | 21.0 | 81 | 0.0 | 87 | 2.9 | 100 | 5.5 | 100 | 0.1 |
| **PFOA** | 64 | 1.4 | 61 | 2.0 | 96 | 0.8 | 91 | 3.9 | 94 | 0.7 |  | 75 | 3.2 | 62 | 0.8 | 88 | 4.5 | 89 | 3.2 | 98 | 2.1 |
| **PFOS** | 84 | 3.5 | 75 | 4.5 | 110 | 11.0 | 110 | 6.7 | 120 | 6.3 |  | 64 | 0.7 | 74 | 1.9 | 82 | 3.0 | 110 | 6.0 | 100 | 7.3 |
| PFPeA | 85 | 1.3 | 98 | 1.3 | 99 | 1.4 | 98 | 2.0 | 94 | 4.1 |  | 96 | 3.7 | 80 | 4.2 | 97 | 5.5 | 110 | 4.6 | 110 | 0.1 |
| Salicylic ac. | 17 | 1.1 | 68 | 0.1 | 38 | 0.9 | 46 | 3.7 | 37 | 0.5 |  | 21 | 2.0 | 54 | 0.4 | 20 | 1.2 | 42 | 3.7 | 17 | 0.7 |
| Terbuthylazine | 84 | 0.9 | 93 | 1.4 | 93 | 0.4 | 96 | 7.1 | 91 | 0.2 |  | 97 | 0.4 | 86 | 0.5 | 100 | 1.9 | 85 | 1.8 | 100 | 0.8 |
| Triclosan | 69 | 2.2 | 96 | 2.0 | 120 | 0.4 | 91 | 4.9 | 99 | 4.1 |  | 120 | 4.5 | 130 | 4.0 | 130 | 0.2 | 89 | 6.4 | 120 | 2.8 |
| **Vildagliptin** | 93 | 3.2 | 63 | 4.7 | 99 | 11.6 | 94 | 4.1 | 120 | 9.2 |  | 110 | 7.0 | 91 | 17.0 | 120 | 4.0 | 100 | 6.3 | 100 | 6.5 |

1. *Bold files represent RR%, the other compounds are represented in E%.*
2. *“-“ indicates that the compound was not recovered*

**PFOS peak**

**PFOS peak**

**PFOS peak**

**PFOS peak**

**PFOS peak**

**Unknown peak**

**PFOS peak**

**Unknown peak**

**Unknown peak**

**Calibrant**

**500 ng/mL**

**Muscle**

**250 ng/g**

**Muscle**

**500 ng/g**

**Liver**

**500 ng/g**

**Liver**

**250 ng/g**

**Liver**

**50 ng/g**

***Figure S-1:*** *PFOS chromatograms in spiked eel liver and muscle, and the 500 ng/mL point of the calibration curve. Liver samples present a secondary peak at T ≈ 13.8 min. which is not a characteristic peak of the PFOS.*

1. * Corresponding author

   Phone: +34-963-543-092 / +34-963-424-216

   E-mail: dyana.vitale@uv.es [↑](#footnote-ref-1)
